# Contactless and Calibration-Free Blood Pressure and Pulse Rate Monitor for Screening and Monitoring of Hypertension: Cross-Sectional Validation Study

**DOI:** 10.2196/57241

**Published:** 2024-08-05

**Authors:** Melissa Kapoor, Blair Holman, Carolyn Cohen

**Affiliations:** 1 Mind over Matter Medtech Ltd London United Kingdom; 2 Element Materials Technology Boulder Louisville, CO United States

**Keywords:** remote photoplethysmography, vital signs, calibration-free blood pressure monitor, medical device, hypertension screening, home blood pressure monitoring, vital, vitals, device, devices, hypertension, hypertensive, cardiovascular, cardiology, heart, blood pressure, monitoring, monitor, mHealth, mobile health, validation

## Abstract

**Background:**

The key to reducing the immense morbidity and mortality burdens of cardiovascular diseases is to help people keep their blood pressure (BP) at safe levels. This requires that more people with hypertension be identified, diagnosed, and given tools to lower their BP. BP monitors are critical to hypertension diagnosis and management. However, there are characteristics of conventional BP monitors (oscillometric cuff sphygmomanometers) that hinder rapid and effective hypertension diagnosis and management. Calibration-free, software-only BP monitors that operate on ubiquitous mobile devices can enable on-demand BP monitoring, overcoming the hardware barriers of conventional BP monitors.

**Objective:**

This study aims to investigate the accuracy of a contactless BP monitor software app for classifying the full range of clinically relevant BPs as hypertensive or nonhypertensive and to evaluate its accuracy for measuring the pulse rate (PR) and BP of people with BPs relevant to stage-1 hypertension.

**Methods:**

The software app, known commercially as Lifelight, was investigated following the data collection and data analysis methodology outlined in International Organization for Standardization (ISO) 81060-2:2018/AMD 1:2020 “Non-invasive Sphygmomanometers—Part 2: Clinical investigation of automated measurement type.” This validation study was conducted by the independent laboratory Element Materials Technology Boulder (formerly Clinimark). The study generated data from 85 people aged 18-85 years with a wide-ranging distribution of BPs specified in ISO 81060-2:2018/AMD 1:2020. At least 20% were required to have Fitzpatrick scale skin tones of 5 or 6 (ie, dark skin tones). The accuracy of the app’s BP measurements was assessed by comparing its BP measurements with measurements made by dual-observer manual auscultation using the same-arm sequential method specified in ISO 81060-2:2018/AMD 1:2020. The accuracy of the app’s PR measurements was assessed by comparing its measurements with concurrent electroencephalography-derived heart rate values.

**Results:**

The app measured PR with an accuracy root-mean-square of 1.3 beats per minute and mean absolute error of 1.1 (SD 0.8) beats per minute. The sensitivity and specificity with which it determined that BPs exceeded the in-clinic systolic threshold for hypertension diagnosis were 70.1% and 71.7%, respectively. These rates are consistent with those reported for conventional BP monitors in a literature review by The National Institute for Health and Care Excellence. The app’s mean error for measuring BP in the range of normotension and stage-1 hypertension (ie, 65/85, 76% of participants) was 6.5 (SD 12.9) mm Hg for systolic BP and 0.4 (SD 10.6) mm Hg for diastolic BP. Mean absolute error was 11.3 (SD 10.0) mm Hg and 8.6 (SD 6.8) mm Hg, respectively.

**Conclusions:**

A calibration-free, software-only medical device was independently tested against ISO 81060-2:2018/AMD 1:2020. The safety and performance demonstrated in this study suggest that this technique could be a potential solution for rapid and scalable screening and management of hypertension.

## Introduction

Cardiovascular disease (CVD) is the largest cause of death worldwide, accounting for approximately 19 million deaths a year [1]. In the European Union, it accounts for almost one-third of all deaths [2], and in England, it accounts for one-quarter of all deaths [3]. Health inequality related to CVD is pronounced, with people living in the most deprived areas of England being more than twice as likely to die from CVD than people in the least deprived areas [4]. In the United States, disparities in CVD prevalence between the richest and poorest populations are not only substantial but growing [5].

High blood pressure (BP) is the leading risk factor for CVD [6]: globally, 54% of strokes and 47% of myocardial infarctions are attributable to hypertension [7]. Therefore, BP is the best single indicator for identifying people at risk of CVD; once someone is diagnosed with hypertension, they can receive clinical support (lifestyle changes and antihypertensive medication) to reduce their risk of CVD. Indeed, BP is also the most important aspect of health for a patient with diagnosed hypertension to try to control (ie, keep at safe levels) to reduce their risk of CVD: every 10 mm Hg reduction in BP down to a systolic BP of 110 mm Hg results in a 17% reduction in coronary heart disease, 27% reduction in stroke, 28% reduction in heart failure, and 13% reduction in all-cause mortality [8].

The large number of deaths caused by CVD plus the high health care and societal costs of CVD events (myocardial infarctions and strokes) are driving a strong global push to identify people with undiagnosed hypertension and then ensure their hypertension is well controlled. In the United States, up to 1 in 8 patients with hypertension may not be diagnosed [9]. In England, 29% of people with hypertension are undiagnosed. This equates to 4.2 million people with undiagnosed hypertension [10]. Nationally, a target has been set to increase the percentage of hypertension cases that are diagnosed to 80% by 2029 [11], a target that will be more challenging to meet than was originally expected because it is estimated that the COVID-19 pandemic prevented or delayed almost 500,000 diagnoses of hypertension across England, Scotland, and Wales [12]. Rapidly identifying millions of people with undiagnosed hypertension requires a highly innovative and rapidly scalable approach. This is particularly true where a patient’s hypertension may be undiagnosed because they are less engaged with conventional health care services, such as NHS Health Checks in England and people without health insurance in the United States.

Novel approaches are needed to ensure patients with diagnosed hypertension control their BP to safe levels (eg, in-clinic systolic BP <140 mm Hg and diastolic BP <90 mm Hg). Rates of BP control achieved using current approaches (typically home BP monitoring [HBPM] using an automated oscillometric BP cuff) are repeatedly shown to be low; in the United States, 43.7% of people with treated hypertension have controlled BP [13]. In the United Kingdom, this rate is lower at approximately 38.1% [14].

HBPM can help improve BP control, especially when it is in combination with cointerventions such as systematic medication titration, patient education, or lifestyle counseling [15]. However, many people with diagnosed hypertension do not own a BP monitor: one UK study found the rate to be not much more than half of treated patients [16]. For many patients who do own a BP monitor, the challenges to using the monitor mean they do not measure their BP according to clinical instructions. These challenges include their inconvenience (bulky size, need to roll up sleeves, and need for regular calibration); their difficulty in operating; and the discomfort that they can cause, particularly to patients with learning difficulties, cognitive impairments, mental illness, or frailty. One study in the United States found that only 38.7% of people with diagnosed hypertension report that they regularly self-monitor their BP [17]. With almost 94 million diagnosed in the United States alone [13,18], there is an opportunity to improve the BP control of many millions of people by overcoming the barriers to BP monitoring created by the hardware nature of conventional BP monitors.

Pulse rate (PR) is another vital sign that can be beneficial to monitor in people with hypertension. The COVID-19 pandemic highlighted the heightened risk of infectious diseases in people with hypertension; a 2.5-fold increase in severity and mortality was observed [19]. It has been shown that presymptomatic infections such as COVID-19 can be detected by regular monitoring of heart rate (HR) [20].

The majority of the world’s population has the equipment needed to use on-demand digital health apps, which could help to reduce health inequalities linked to access to and attitudes or behaviors toward specialist medical equipment: in the United States, it is estimated that 92% of the population owned a smartphone in 2023 [21]. Smartphone ownership is also increasing in low-resource countries; it is predicted to reach 75% in India by 2026 [22]. Software-only BP monitors that do not require calibration have particular promise for enabling high-volume hypertension screening that is currently unfeasible with conventional BP monitors or innovative BP monitors that require initial user calibration using separate hardware. Contactless BP monitors that require no specialist equipment could potentially improve adherence to HBPM. It is therefore reasonable to expect that software-only BP monitors can enable earlier and better detection of hypertension and improve rates of BP control among people with diagnosed hypertension, leading to better patient outcomes.

Element Materials Technology Boulder (formerly Clinimark) completed a clinical investigation of the Lifelight software-only BP monitor, commissioned by the app’s manufacturer. The study aimed to assess the app’s accuracy in measuring PR and BP.

## Methods

### Overview

The procedure, data collection methods, and data analysis methods of the validation study follow applicable sections of the following: International Standard International Organization for Standardization (ISO) 81060-2:2018/AMD 1:2020 “Non-invasive sphygmomanometers—Part 2: Clinical investigation of intermittent automated measurement type,” where relevant to the device under investigation; ISO 14155:2020 “Clinical investigation of medical devices for human participants—Good clinical practice”; Medical Device Regulation European Union 2017/745; and Code of Federal Regulations for Nonsignificant Risk Devices.

### Ethical Considerations

The study was performed in accordance with the Declaration of Helsinki, 21 CFR 50, and 21 CFR 812 for nonsignificant risk device study investigations. The study only commenced once approval was received from the Independent Review Board (IRB) for testing through Salus IRB (project 2022-513; approved on March 6, 2023).

### Participants and Recruitment

Participants were volunteers aged 18 to 85 years old who received an invitation from Element Materials Technology Boulder via phone or email to take part in the study. Some of the participants were known to Element to be suitable based on their BP values in previous studies. Since ISO 81060-2:2018 prescribes minimum participation levels at the extremes of the BP range (Table 1), recruitment was organized such that most of the potential participants at the hypotensive and high hypertensive ends of the range were invited first. These then either helped to fill most of the required allocations at the extremes, or the adjacent categories if their BP measurements on the day (using the baseline reference auscultation) put them in the next higher BP category (hypotensive) or next lower category (hypertensive). Thus, participants generally were included from the extremes of the range toward the normotensive center of the required distribution.

**Table 1:** Required blood pressure (BP) distribution of the 85 participants in the laboratory-based, cross-sectional validation study of the software-only and calibration-free BP monitor.

BP range	Required percentage of the 85 study participants
Systolic BP ≤100 mm Hg (hypotension)	≥5%
Systolic BP ≥140 mm Hg (hypertension)	≥20%
Systolic BP ≥160 mm Hg (high hypertension)	≥5%
Diastolic BP ≤60 mm Hg (hypotension)	≥5%
Diastolic BP ≥85 mm Hg (hypertension)	≥20%
Diastolic BP ≥100 mm Hg (high hypertension)	≥5%

When potential participants arrived in the Element laboratory, the procedure was explained to them, and an IRB-approved informed consent form was provided to them. The informed consent outlined study designs as well as the rights and obligations of the participant. The study staff was available to answer any questions about this study or the form. Participants who were satisfied that all of their questions had been satisfactorily answered, who completed the informed consent and health screening, and who met all of the inclusion criteria and none of the exclusion criteria were enrolled in the study. Exclusion criteria were as follows: medically unsuitable for participation at the time of visit (the principal investigator or clinician used their medical discretion to not enroll participants if the participant’s self-reported condition would compromise participant safety if they were enrolled); any heart dysrhythmia (except respiratory sinus arrhythmia) as confirmed with 3-lead electroencephalography (ECG); compromised circulation or peripheral vascular disease; clotting disorder; excessive facial hair; or conditions that affect the skin, such as anemia, jaundice, rosacea, psoriasis, acute acne, and erythropoietic protoporphyria. Female participants who were pregnant or trying to get pregnant were also excluded. The app is currently contraindicated for these people.

Where a participant would not add to the remaining skin tone or BP sample size requirements, they also were not enrolled. This was done to avoid further diluting the percentages of participants with darker skin tones and low and very high BPs.

Study data were deidentified and a participant number was used for the day of the test along with participant demographics. Records identifying participants’ names (informed consent and health forms) were kept in a secured location with either a locked file or a locked door.

Participants could choose to withdraw themselves from the study without prejudice or they could be withdrawn by study investigators for predetermined reasons. One predetermined reason for withdrawing a participant was determined after their study participation had taken place: it was determined that their removal would help the study to reach its skin tone and BP sample size requirements sooner and without unnecessarily increasing the overall sample size of the study, which would incur extra cost. In this case, relevant participants were withdrawn in a last-one-in, last-one-out approach. Data excluded from the analysis were documented with justifications.

The only direct benefit to participating in this study was being a paid volunteer. At the end of their study participation in the laboratory, participants were each paid US $100 for participating.

### Study Procedures

The study was conducted from May 18, 2023, to August 3, 2023, in the Element Materials Technology laboratory in Louisville, Colorado, United States, in accordance with the study procedure. Study notes were made to describe the conditions of each test as well as deviations, device issues, and any adverse events. There was no additional follow-up with the participants.

The same-arm sequential method was used to assess the app’s accuracy for measuring BP against the dual auscultation reference data. The reference BP measurements were made by 2 trained observers using a digital sphygmomanometer (with a maximum error of SD 1 mm Hg per NIST traceable calibration verification) with a released BP cuff and a dual auscultatory stethoscope to listen to the Korotkoff sounds at the brachial artery of each participant’s bare left arm. Each observer’s recording of observations of the reference sphygmomanometer was not visible to the other observer and neither observer could see the measurements recorded by the app. The actual reference BP measurements are the average of each consecutive pair of reference BP recordings (ie, the recording made before a given measurement of BP using the app and the measurement made afterward). These reference BP measurements determine which BP band each measurement set contributes to (hypotension, normotension, stage-1 hypertension, or stage-2/3 hypertension). The participant’s age, sex, and height data were entered into the app before measurements were taken, as the device uses these biometrics in addition to calculated signal features in the machine learning algorithms for BP [23]. The frame rate of the smart device on which the app was run was 30 frames per second and the image resolution was 1080 pixels. The app does not produce measurements if the frame rate drops. There are a minimum number of pixels in the region of interest taken from the midface. Participants were seated in front of 2 photographic quality LED light panels.

After the participants had rested in the seated position for at least 5 minutes with legs uncrossed; feet flat on the floor; and back, elbow, and forearm supported, 1 or 2 initial baseline reference BP recordings were taken. Then, up to 8 pairs of reference and app recordings (starting and ending with reference recordings) were taken sequentially to obtain a minimum of 3 valid paired reference and app BP measurements. At least 60 seconds elapsed between each BP determination.

Any pair of observers’ reference BP recordings with a difference greater than 4 mm Hg were excluded and additional pairs of measurements (up to 8 in total) were taken to ensure that no more than 10% of the participants had fewer than 3 valid pairs of BP readings.

Simultaneous to the BP measurements, a Food and Drug Administration–cleared ECG HR monitor (GE Healthcare S5 Compact Monitor) recording HR at 0.2 Hz was used as the reference for app-derived PR measurements. This ECG recording was continuous; reference measurements were the average over the 60-second window that the app was running simultaneously.

### Sample Size

The sample size calculation for the full dataset collected in this study is defined by ISO 81060-2:2018/AMD 1:2020. The requirement for 85 participants originated from the early work of the ANSI/AAMI BP committee dating from 1987 [24].

The distribution of reference BP measurements for the 85 participants is also defined by ISO 81060-2:2018/AMD 1:2020 as presented in Table 1.

Additionally, the ISO standard requires that at least 30% of participants are male and at least 30% are female. We additionally set the requirement that at least 20% of the study population should have a Fitzpatrick score of 5 or 6 (as assessed using the Mexameter MX 18 Melanin Density meter Photonova). Although no standard related to BP monitors or remote photoplethysmography (rPPG)–based medical technologies have requirements on the skin tone distribution of validation study participants, the Food and Drug Administration has issued guidance that pulse oximeters (a PPG technology) should be validated on a population where at least 15% of participants have dark skin tones [25]. We therefore set the requirement for at least 20% of the participants in our validation study to have Fitzpatrick skin tones of 5 or 6 to exceed the requirement for pulse oximeters. Our protocol allowed us to recruit up to 200 volunteers in order to secure the requisite data for analysis from 85 participants.

For the PR analysis, this paper reports on the full dataset collected from study participants (ie, data from 85 participants meeting all skin tone and BP distribution requirements). For BP, we provide two sets of analyses: (1) analyses related to the use case of hypertension screening, which is relevant to people with any BP (from hypotension through to high hypertension), and (2) analyses related to the use case of HBPM by people with stage-1 hypertension (BP up to 160/100 mm Hg). Therefore, the full dataset collected from study participants in the validation study is used for analyses related to the hypertension screening use case (ie, data from 85 participants meeting all skin tone and BP distribution requirements). However, for the analyses related to HBPM, only the normotensive and stage-1 hypertensive BP measurements are included, that is, reference systolic BP data points of 100-159 mm Hg and reference diastolic BP data points of 60-99 mm Hg. Therefore, the analyses related to HBPM reported in this paper are made using data from fewer than 85 participants.

### Data Analysis

All statistical analyses were performed using the Python object-oriented programming language. We did not perform imputation of missing or implausible data, and any missing, implausible, or problematic readings were excluded from the analysis. Where the app was unable to detect the participant’s face during the measurement period, this was recorded in the care report form as a device deficiency and the measurements were not analyzed. Data for a given participant were considered valid if the reference systolic BP determinations did not differ by more than 12 mm Hg and the reference diastolic BP determinations did not differ by more than 8 mm Hg over the range of readings once the participant had settled.

This paper reports the mean error with SD and also mean absolute error (MAE) with associated SD of the app’s BP measurements in cases where BP is either normotensive or stage-1 hypertensive. Mean error and SD comprise the Criterion 1 performance requirements stated in ISO 81060-2:2018/AMD 1:2020, which are the clinically sought-after performance metrics for these 2 use cases.

This paper also reports the app’s performance for measuring BP in terms of its accuracy in classifying a given measurement set (simultaneous systolic and diastolic BP measurements) as either normotensive or hypertensive. A hypertensive systolic BP measurement (≥ 140 mm Hg) or a hypertensive diastolic BP measurement (≥ 85 mm Hg) can determine a measurement set to be hypertensive. This performance metric is particularly relevant to the use case of screening people with any level of BP for hypertension.

For PR, the app’s PR measurements were compared with the reference 3-lead, ECG-derived HR measurements averaged over the same 60-second period. The app’s performance in this paper is reported as accuracy root-mean-square (A_rms_). The mean bias with SD, MAE with associated SD, and *R*^2^ values are also reported.

## Results

A total of 129 volunteers were screened and enrolled in the study in order to generate data meeting the requirements of ISO 81060-2:2018/AMD 1:2020. No patients were excluded before recruiting these 129 patients because the Element Materials Technology independent laboratory targeted invitations at those people from their existing test panel who they had high confidence would meet all of the inclusion criteria and none of the exclusion criteria. There were no serious adverse events or serious adverse device effects during the study. There were no observed device deficiencies that could have led to serious adverse device effects.

Table 2 presents the demographic distributions of the 85 participants whose data progressed to the PR analyses and BP classification (hypertension screening) analyses. A total of 44 participants were enrolled in the study but subsequently withdrawn because of device deficiencies (n=40—on all of these occasions, the device deficiency was that the app was unable to produce at least 2 BP measurements within 8 attempts), a minor adverse event (unrelated to the app; n=1—this participant was also withdrawn because of device deficiency), participant noncompliance with study procedures (n=1), observers could not determine reference BP measurements (n=1), and the participant would not progress the study closer to reaching its BP distribution requirements (n=2).

**Table 2:** Demographics of the 85 study participants providing data for assessment of the accuracy of the software-only blood pressure (BP) monitor for measuring pulse rate and screening for hypertension in the laboratory-based validation study.

Demographic factor (n=85)			Value
**Sex, n (%)**			
	Male	28 (33)	
	Female	57 (67)	
**Age (years), mean (range)**			47.8 (20-77)
Systolic BP (mm Hg; determined from baseline reference measurement), mean (range)			124 (81-192)
Diastolic BP (mm Hg; determined from baseline reference measurement), mean (range)			78 (48-107)
**Race (participants could report more than 1 race), n (%)**			
	American Indian or Alaskan Native	3 (4)	
	Asian	11 (13)	
	Black or African American	9 (11)	
	White	64 (75)	
	Other	3 (4)	
**Ethnicity, n (%)**			
	Hispanic	9 (11)	
	Non-Hispanic	76 (89)	
**Skin tone, n (%)**			
	Fitzpatrick 5 or 6	17 (20)	
	Fitzpatrick ≤4	68 (80)	

The detailed breakdown of BP distributions for these 85 participants is shown in Table 3. These distributions meet the requirements of ISO 81060-2:2018/AMD 1:2020. Hypotension is defined as systolic BP ≤100 mm Hg and diastolic BP ≤60 mm Hg. Stage-1 hypertension is defined as systolic BP ≥140 mm Hg but <160 mm Hg and diastolic BP ≥85 mm Hg but <100 mm Hg. Stage-2/3 hypertension is defined as systolic BP ≥160 mm Hg and diastolic BP ≥100 mm Hg.

**Table 3:** Systolic and diastolic blood pressure (BP) distributions of the 85 participants in the laboratory-based, cross-sectional validation study of the software-only and calibration-free BP monitor.

	Hypotensive, n (%)	Stage-1 hypertensive, n (%)	Stage-2/3 hypertensive, n (%)
Systolic BP	11 (13)	21 (25)	4 (5)
Diastolic BP	7 (8)	30 (35)	5 (6)

Once measurements falling outside the normotensive and stage-1 hypertensive ranges were excluded, 185 measurements from 65 participants were used to generate the results related to HBPM. The demographic distributions of these 65 participants are shown in Table 4. The distributions of the normotensive/Stage 1 hypertensive reference systolic and diastolic BP measurements from these 65 participants are shown in Table 5.

**Table 4:** Demographics of the 65 participants from the laboratory-based validation study of the software-only blood pressure (BP) monitor who had BPs relevant to the home BP monitoring use case, that is, participants providing normotensive or stage-1 hypertensive measurements only.

Demographic factor (n=65)			Value
**Sex, n (%)**			
	Male	26 (40)	
	Female	39 (60)	
Age (years), mean (range)			49.4 (20-72)
Systolic BP (mm Hg; determined from baseline reference measurement), mean (range)			127 (100-159)
Diastolic BP (mm Hg, determined from baseline reference measurement), mean (range)			81 (62-100)
**Race (participants could report more than one race), n (%)**			
	American Indian/ Alaskan Native	2 (3)	
	Asian	4 (6)	
	Black / African-American	5 (8)	
	White	51 (78)	
	Other	3 (5)	
**Ethnicity, n (%)**			
	Hispanic	6 (9)	
	Non-Hispanic	59 (91)	
**Skin tone, n (%)**			
	Fitzpatrick 5 or 6	11 (17)	
	Fitzpatrick ≤4	54 (83)	

**Table 5:** Systolic and diastolic blood pressure (BP) distributions of the 65 participants from the laboratory-based validation study of the software-only BP monitor who had BPs relevant to the home BP monitoring use case, that is, participants providing normotensive or stage-1 hypertensive measurements only.

	Normotensive, n (%)	Stage-1 hypertensive, n (%)
Systolic BP	49 (75)	16 (25)
Diastolic BP	42 (65)	23 (35)

**Figure 1:**
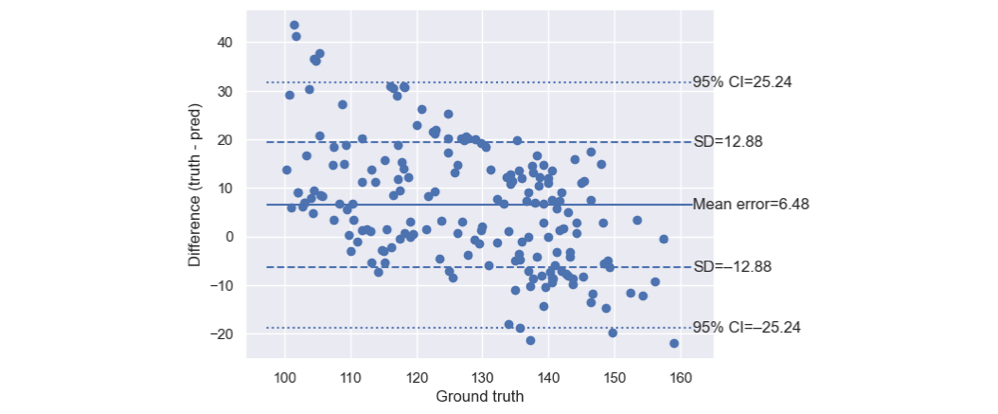
Bland-Altman plot of the systolic blood pressure measurements made by the software-only and calibration-free blood pressure monitor in its laboratory-based cross-sectional validation study on the 65 study participants who had blood pressures relevant to the home blood pressure monitoring use case, that is, participants providing normotensive or stage-1 hypertensive measurements only. The “ground-truth” systolic blood pressures are the systolic blood pressure measurements made by the concurrent dual auscultation reference.

**Figure 2:**
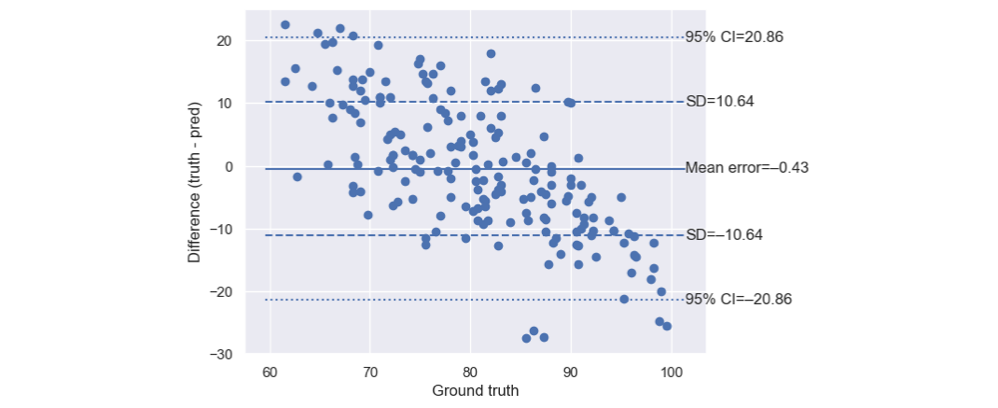
Bland-Altman plot of the diastolic blood pressure measurements made by the software-only and calibration-free blood pressure monitor in its laboratory-based cross-sectional validation study on the 65 study participants who had blood pressures relevant to the home blood pressure monitoring use case, that is, participants providing normotensive or stage-1 hypertensive measurements only. The “ground-truth” diastolic blood pressures are the diastolic blood pressure measurements made by the concurrent dual auscultation reference.

As shown in Figure 1, the mean error of the app’s measurements of systolic BP was 6.5 (SD 12.9) mm Hg. The MAE was 11.3 (SD 10.0) mm Hg. Figure 2 shows that the app’s mean error for measuring diastolic BP was 0.4 (SD 10.6) mm Hg. The MAE was 8.6 (SD 6.8) mm Hg.

**Figure 3:**
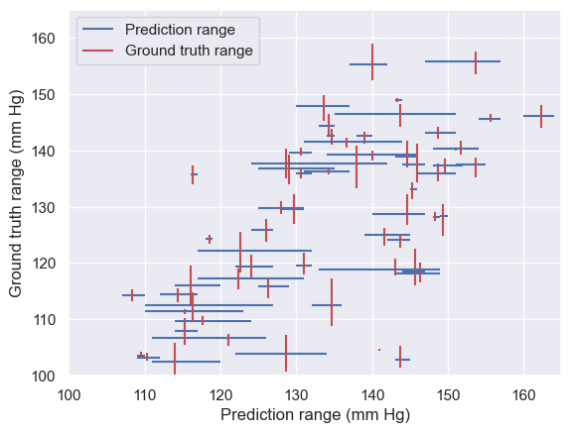
Comparison of the ranges across repeated readings of the dual auscultation reference “ground-truth” systolic blood pressure measurements and the systolic blood pressure measurements made by the software-only blood pressure monitor for each of the 65 cross-sectional validation study participants who had blood pressures relevant to the home blood pressure monitoring use case, that is, participants providing normotensive or stage-1 hypertensive measurements only.

To provide insight into the app’s intrasubject accuracy and reliability, the ranges of ground truth and app measurements across the repeat readings for individual participants are compared in Figure 3. This plot shows that, for some participants, the app repeatedly overestimated or underestimated BP across the repeat readings. The plot also shows that the app had a larger range of repeat systolic BP measurements than the reference manual sphygmomanometry ground truth for some participants, whereas for other participants, the app had a smaller range. Overall, however, the intrasubject variation of the app’s BP measurements is greater than the variation of the reference method. There is no discernible pattern to the app’s intrasubject measurement ranges, suggesting that the errors are randomly distributed across this BP range.

**Figure 4:**
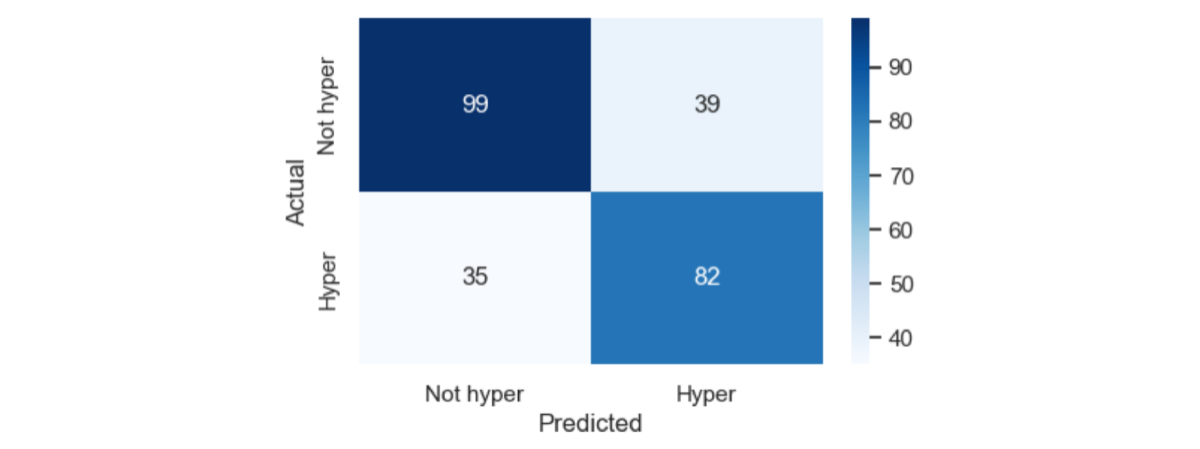
Confusion matrix for the software-only and calibration-free blood pressure monitor for classifying each of the 85 participants in the cross-sectional validation study as hypertensive or not. The “ground-truth” hypertension status is determined by whether both or either the systolic and diastolic blood pressure measurements made by the concurrent dual auscultation reference method fall within hypertensive ranges or not.

The app correctly classified 70.1% of hypertensive systolic measurements (which could be from people with either stage-1 hypertensive or stage-2/3 hypertensive BP measurements) and 71.7% of nonhypertensive systolic measurements (which could be from people with either normotensive or hypotensive BP measurements). The confusion matrix for these classification results is shown in Figure 4.

**Figure 5:**
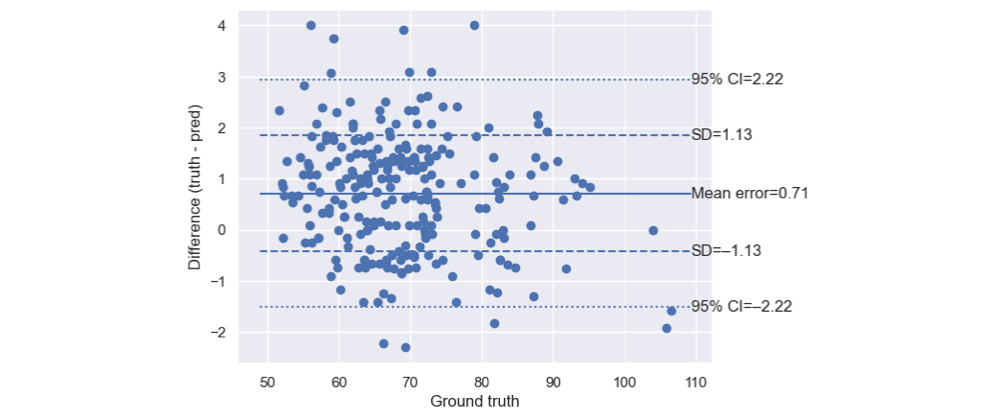
Bland-Altman plot of the pulse rate measurements made by the software-only and calibration-free blood pressure monitor in its laboratory-based cross-sectional validation study on 85 study participants, as compared with the “ground-truth” heart rate measurements made by the concurrent electroencephalography reference.

As shown in Figure 5, the mean bias of the app’s measurements of PR across measurements from all 85 participants was 0.7 (SD 1.1) beats per minute (bpm). The MAE was 1.1 (SD 0.8) bpm. The A_rms_ was 1.3 bpm.

## Discussion

Lifelight is a contactless and calibration-free BP and PR monitor that works on standard smartphones and tablet devices. A validation study adhering to relevant ISO standards was conducted on the app by the Element Materials Technology laboratory (ie, independently of the manufacturer).

This study has shown that the app measures PR with A_rms_ of 1.3 bpm. For patients with normotensive or stage-1 hypertensive BP, the app had a mean error of 6.5 (SD 12.9) mm Hg for measuring systolic BP and 0.4 (SD 10.6) mm Hg for measuring diastolic BP. Therefore, the app appears to perform in line with the state of the art in terms of absolute accuracy; a meta-analysis of regulated state-of-the-art BP monitors (devices that measure BP using contact-based PPG, eg, bracelets, or the volume-clamp method or tonometry at the finger) reported the mean error of these devices for measuring systolic BP (across the full range) to be 6.7 (SD 15.3) mm Hg and 5.5 (SD 8.9) mm Hg for measuring diastolic BP [26]. With regard to the accuracy of the app for distinguishing hypertension from nonhypertension, it was found to have 70.1% sensitivity and 71.7% specificity when assessed across the full range of BP values. These values fall within the ranges reported by the studies included in the National Institute for Health and Care Excellence “Hypertension in adults: diagnosis and management - Evidence review for diagnosis 2019” (Guideline NG136): the in-clinic sensitivities of the clinical studies included in the National Institute for Health and Care Excellence’s evidence review range from 59% to 89.3%, and the specificities range from 41.4% to 81% [27].

It is relevant to consider these accuracy results in light of the accuracy of the current standard of care, which are automated oscillometric BP cuffs and monitors. Automated oscillometric cuffs have inherent errors mostly associated with the empirical algorithms used to derive BP from waveform pulsations. Moreover, it is estimated that systematic errors due to uncalibrated BP monitors account for 28% of cases of undetected hypertension and 32% of false diagnoses of hypertension [28]; systematic error of up to 9 mm Hg can occur within 3 months of calibration [29]. There is differing evidence in the published literature about the state of BP cuffs and monitors owned by individuals in the community. The ACCU-RATE study in England found that 76% of BP cuff and monitor systems owned by primary care patients diagnosed with hypertension were accurate to within 3 mm Hg over the range 0 and 270/300 mm Hg, and only 5% recorded a measurement more than 5 mm Hg different from the ground truth. The largest error recorded by one of these devices was 11.4 mm Hg [30]. On the other hand, a study in Canada found these types of devices to generally be less accurate: 69%, 29% and 7% of HBPM devices sourced from individual owners produced BP measurements with errors of ≥5, 10, and 15 mm Hg, respectively [31].

Compounded with these integral sources of error of BP cuffs and monitors are the (generally larger) errors that occur when these devices are incorrectly operated; one study found that only 62.8% of hypertensive patients place the cuff correctly and only 65.2% place it on a bare arm—a misuse that can cause BP measurement errors of up to 50 mm Hg [32,33]. Another source of major concern is the number of BP cuffs and monitors available on the market that are not validated; one study in Australia found that only 18.3% of upper-arm cuff devices available for purchase on the internet were validated [34]. It should be noted that these sources of error and concern are not relevant to devices that measure central BP and so these devices are typically used in hypertension research instead of BP cuffs.

Strengths of this study that future studies on the app should aim to emulate include how well subject positioning was controlled with respect to their height, back angle, and head angle relative to the camera; the consistency of the lighting through the use of 2 photographic quality LED light panels with adjustable output; and the use of continuous ECG to ensure concurrent signal was available for the PR analyses, eliminating the risk of physiological variation impacting the comparison.

A weakness of this study was that overrecruitment was required to meet the demographic and BP distribution requirements, particularly because of the app’s “device deficiencies,” that is, the inability to make a PR and BP measurement in some participants; where the rPPG signal quality does not meet prespecified thresholds, the app returns no PR and BP measurements.

A limitation of this study is that all of the results reported in this paper relate to the use of the app on people for whom the app is not currently contraindicated. If it were used on people for whom the app is contraindicated, including people with vascular disease or atrial fibrillation, different performance statistics may apply. Not everyone who has diseases like these is aware they have them.

Another limitation is that the results may not reflect the accuracy of the app under real-world conditions; it is unrealistic to assume that patients would set up photographic quality LED light panels when using them in a real-world situation. A lower real-world accuracy may still be consistent with clinical use cases because the calibration-free and software-only nature of the app means it can enable BP measurements in circumstances where they otherwise would not occur or might be made using unregulated methods. For example, a home-based, software-only method may engage individuals who are disengaged from traditional hypertension screening approaches, such as NHS Health Checks. This could facilitate the shift from in-person health checks to digital health checks by providing a method for measuring BP with proven accuracy and therefore predictable impacts at the population level. The alternative would be patients producing BP measurements of unknown provenance, which could have unpredictable implications and impacts on health care systems, given how many unregulated BP monitors are available on the market today [34]. Another relevant clinical use case is HBPM in low-resource contexts where traditional health care resources and services are unavailable, inaccessible, or extremely limited. For all clinical use cases, the random nature of the app’s current intrasubject variation means that it may be pertinent for triage-type clinical decisions to be made using multiple measurements instead of one measurement only. Clinical decisions can then be confirmed using standard-of-care methods.

In summary, this validation study suggests that calibration-free and contactless technologies that only require ubiquitous equipment (eg, standard smartphones) could potentially in future be a means to more rapid and scalable hypertension screening and more prevalent HBPM. Globally, 41% of women and 51% of men with hypertension are not diagnosed, and only 21% of people with hypertension have it under control [35,36]. This means that more than a billion people could benefit from a more accessible and convenient method for measuring their BP. This could lead to a significant reduction in global health burden.

## Abbreviations

Arms: accuracy root-mean-square
BP: blood pressure
bpm: beats per minute
CVD: cardiovascular disease
ECG: electroencephalography
HBPM: home blood pressure monitoring
HR: heart rate
IRB: Independent Review Board
ISO: International Organization for Standardization
MAE: mean absolute error
PR: pulse rate
rPPG: remote photoplethysmography

## Data Availability

The datasets generated or analyzed during this study are not publicly available due to their commercially sensitive nature, as mandated by Xim Limited’s grant funders and investors, but are available from the corresponding author on reasonable request.

## References

[1] Roth GA, Mensah GA, Johnson CO, Addolorato G, Ammirati E, Baddour LM, Barengo NC, Beaton AZ, Benjamin EJ, Benziger CP, Bonny A, Brauer M, Brodmann M, Cahill TJ, Carapetis J, Catapano AL, Chugh SS, Cooper LT, Coresh J, Criqui M, DeCleene N, Eagle KA, Emmons-Bell S, Feigin VL, Fernández-Solà J, Fowkes G, Gakidou E, Grundy SM, He FJ, Howard G, Hu F, Inker L, Karthikeyan G, Kassebaum N, Koroshetz W, Lavie C, Lloyd-Jones D, Lu HS, Mirijello A, Temesgen AM, Mokdad A, Moran AE, Muntner P, Narula J, Neal B, Ntsekhe M, Moraes de Oliveira G, Otto C, Owolabi M, Pratt M, Rajagopalan S, Reitsma M, Ribeiro ALP, Rigotti N, Rodgers A, Sable C, Shakil S, Sliwa-Hahnle K, Stark B, Sundström J, Timpel P, Tleyjeh IM, Valgimigli M, Vos T, Whelton PK, Yacoub M, Zuhlke L, Murray C, Fuster V (2020). Global burden of cardiovascular diseases and risk factors, 1990-2019: update from the GBD 2019 study. J Am Coll Cardiol.

[2] Eurostat (2020). Cardiovascular diseases statistics. eurostat.

[3] Raleigh V, Jefferies D, Wellings D (2022). Cardiovascular disease in England. The King's Fund.

[4] (2023). Inequalities in mortality involving common physical health conditions, England. Office for National Statistics.

[5] Abdalla SM, Yu S, Galea S (2020). Trends in cardiovascular disease prevalence by income level in the United States. JAMA Netw Open.

[6] Fuchs FD, Whelton PK (2020). High blood pressure and cardiovascular disease. Hypertension.

[7] Lawes CMM, Vander Hoorn S, Rodgers A, International Society of Hypertension (2008). Global burden of blood-pressure-related disease, 2001. Lancet.

[8] Ettehad D, Emdin CA, Kiran A, Rahimi K (2016). Blood pressure lowering for cardiovascular disease - authors' reply. Lancet.

[9] Ciemins EL, Ritchey MD, Joshi VV, Loustalot F, Hannan J, Cuddeback JK (2018). Application of a tool to identify undiagnosed hypertension - United States, 2016. MMWR Morb Mortal Wkly Rep.

[10] (2023). Risk factors for undiagnosed high blood pressure in England. Office for National Statistics.

[11] (2019). Ambitions set to address major causes of cardiovascular disease. Public Health England and NHS England.

[12] Dale CE, Takhar R, Carragher R, Katsoulis M, Torabi F, Duffield S, Kent S, Mueller T, Kurdi A, Le Anh TN, McTaggart S, Abbasizanjani H, Hollings S, Scourfield A, Lyons RA, Griffiths R, Lyons J, Davies G, Harris D, Handy A, Mizani MA, Tomlinson C, Thygesen JH, Ashworth M, Denaxas S, Banerjee A, Sterne JAC, Brown P, Bullard I, Priedon R, Mamas MA, Slee A, Lorgelly P, Pirmohamed M, Khunti K, Morris AD, Sudlow C, Akbari A, Bennie M, Sattar N, Sofat R (2023). The impact of the COVID-19 pandemic on cardiovascular disease prevention and management. Nat Med.

[13] Muntner P, Hardy ST, Fine LJ, Jaeger BC, Wozniak G, Levitan EB, Colantonio LD (2020). Trends in blood pressure control among us adults with hypertension, 1999-2000 to 2017-2018. JAMA.

[14] Tapela N, Collister J, Clifton L, Turnbull I, Rahimi K, Hunter DJ (2021). Prevalence and determinants of hypertension control among almost 100 000 treated adults in the UK. Open Heart.

[15] Tucker KL, Sheppard JP, Stevens R, Bosworth HB, Bove A, Bray EP, Earle K, George J, Godwin M, Green BB, Hebert P, Hobbs FDR, Kantola I, Kerry SM, Leiva A, Magid DJ, Mant J, Margolis KL, McKinstry B, McLaughlin MA, Omboni S, Ogedegbe O, Parati G, Qamar N, Tabaei BP, Varis J, Verberk WJ, Wakefield BJ, McManus RJ (2017). Self-monitoring of blood pressure in hypertension: a systematic review and individual patient data meta-analysis. PLoS Med.

[16] Anbarasan T, Rogers A, Rorie DA, Grieve JWK, Flynn RWV, MacDonald TM, Mackenzie IS (2022). Factors influencing home blood pressure monitor ownership in a large clinical trial. J Hum Hypertens.

[17] Ostchega Y, Zhang G, Kit BK, Nwankwo T (2017). Factors associated with home blood pressure monitoring among US adults: national health and nutrition examination survey, 2011-2014. Am J Hypertens.

[18] Tsao CW, Aday AW, Almarzooq ZI, Alonso A, Beaton AZ, Bittencourt MS, Boehme AK, Buxton AE, Carson AP, Commodore-Mensah Y, Elkind MSV, Evenson KR, Eze-Nliam C, Ferguson JF, Generoso G, Ho JE, Kalani R, Khan SS, Kissela BM, Knutson KL, Levine DA, Lewis TT, Liu J, Loop MS, Ma J, Mussolino ME, Navaneethan SD, Perak AM, Poudel R, Rezk-Hanna M, Roth GA, Schroeder EB, Shah SH, Thacker EL, VanWagner LB, Virani SS, Voecks JH, Wang N, Yaffe K, Martin SS (2022). Heart disease and stroke statistics-2022 update: a report from the American heart association. Circulation.

[19] Lippi G, Wong J, Henry BM (2020). Hypertension in patients with coronavirus disease 2019 (COVID-19): a pooled analysis. Pol Arch Intern Med.

[20] Mishra T, Wang M, Metwally AA, Bogu GK, Brooks AW, Bahmani A, Alavi A, Celli A, Higgs E, Dagan-Rosenfeld O, Fay B, Kirkpatrick S, Kellogg R, Gibson M, Wang T, Hunting EM, Mamic P, Ganz AB, Rolnik B, Li X, Snyder MP (2020). Pre-symptomatic detection of COVID-19 from smartwatch data. Nat Biomed Eng.

[21] (2023). Cell phone statistics 2024. Consumer Affairs.

[22] (2022). TMT predictions. Deloitte.

[23] van Putten LD, Bamford KE, Veleslavov I, Wegerif S (2024). From video to vital signs: using personal device cameras to measure pulse rate and predict blood pressure using explainable AI. Discov Appl Sci.

[24] (1993). American National Standard for Electronic or Automated Sphygmomanometers: ANSI/AAMI SP10-1987.

[25] FDA (2013). Pulse oximeters - premarket notification submissions [510(k)s]: guidance for industry and food and drug administration staff. US Food & Drug Administration.

[26] Heiden E, Jones T, Brogaard Maczka A, Kapoor M, Chauhan M, Wiffen L, Barham H, Holland J, Saxena M, Wegerif S, Brown T, Lomax M, Massey H, Rostami S, Pearce L, Chauhan A (2022). Measurement of vital signs using lifelight remote photoplethysmography: results of the VISION-D and VISION-V observational studies. JMIR Form Res.

[27] (2019). Hypertension in adults: diagnosis and management - evidence review for diagnosis NICE guideline NG136. NICE.

[28] Turner MJ, Irwig L, Bune AJ, Kam PC, Baker AB (2006). Lack of sphygmomanometer calibration causes over- and under-detection of hypertension: a computer simulation study. J Hypertens.

[29] Abderahman HN, Dajani HR, Bolic M, Groza V (2016). An integrated system to compensate for temperature drift and ageing in non-invasive blood pressure measurement.

[30] Hodgkinson JA, Lee M, Milner S, Bradburn P, Stevens R, Hobbs FR, Koshiaris C, Grant S, Mant J, McManus RJ (2020). Accuracy of blood-pressure monitors owned by patients with hypertension (ACCU-RATE study): a cross-sectional, observational study in central England. Br J Gen Pract.

[31] Ringrose J, Polley G, McLean D, Thompson A, Morales F, Padwal R (2017). An assessment of the accuracy of home blood pressure monitors when used in device owners. Am J Hypertens.

[32] Flacco ME, Manzoli L, Bucci M, Capasso L, Comparcini D, Simonetti V, Gualano MR, Nocciolini M, D'Amario C, Cicolini G (2015). Uneven accuracy of home blood pressure measurement: a multicentric survey. J Clin Hypertens (Greenwich).

[33] Whelton SP, Ebinger J, Yang E (2023). Why is cuff size so important and other factors that affect accurate blood pressure measurement. American College of Cardiology.

[34] Picone DS, Deshpande RA, Schultz MG, Fonseca R, Campbell NR, Delles C, Hecht Olsen M, Schutte AE, Stergiou G, Padwal R, Zhang X, Sharman JE (2020). Nonvalidated home blood pressure devices dominate the online marketplace in Australia: major implications for cardiovascular risk management. Hypertension.

[35] Zhou B, Carrillo-Larco RM, Riley LM, Paciorek CJ, Stevens GA, Gregg EW, Bennett JE, Solomon B, Singleton RK, Sophiea MK, Iurilli ML, Lhoste VP, Cowan MJ, Savin S, Woodward M, Balanova Y, Cifkova R, Damasceno A, Elliott P (2021). Worldwide trends in hypertension prevalence and progress in treatment and control from 1990 to 2019: a pooled analysis of 1201 population-representative studies with 104 million participants. Lancet.

[36] (2023). Global report on hypertension: the race against a silent killer. World Health Organisation.

